# Biomarkers in the Era of Precision Oncology

**DOI:** 10.3390/cancers15061782

**Published:** 2023-03-15

**Authors:** Constantin N. Baxevanis

**Affiliations:** Cancer Immunology and Immunotherapy Center, Cancer Research Center, Saint Savas Cancer Hospital, 11522 Athens, Greece; costas.baxevanis@gmail.com; Tel.: +30-2106409380

Cancer heterogeneity provides a formidable obstacle to optimizing clinical protocols to achieve durable clinical responses. Notwithstanding the progress in science and technology which improved the molecular characterization of cancer, the oncogenic pathways underlying its progression are not adequately explored and, therefore, their therapeutic targeting is ineffective. The absence of reliable biomarkers for the early detection and progression monitoring results in the generation of complex cancer-related molecular pathways negatively impact anticancer immune-mediated responses. As a result, immunotherapy-based therapeutic modalities alone or in combination with other standard or targeted therapies provide limited clinical benefits for patients. Therefore, it is imperative to discover biomarkers suitable for selecting patients most likely to benefit from therapies. Precision oncology is mostly based on the high-throughput molecular profiling of tumors which allows for the identification of genomic modifications spotlighting appropriate research and therapeutic targeting. A comprehensive understanding of the landscape of genetic alterations in tumors will certainly further advance our understanding of the dynamic interactions between tumor cells and immune subpopulations, resulting in the development of rational combinatorial therapies. This Special Issue aimed to review the impact of immune and molecular profiling for the identification of predictive and prognostic biomarkers in various types of malignant diseases and their relevance in terms of clinical response or resistance to therapeutic treatments.

So far, prostate cancer (PCa) lacks adequate biomarkers for prognosis, which would be most useful to guide the better design of clinical therapeutic treatments to be applied in the appropriate clinical setting and improve clinical benefits for patients. In their study, Bonattera et al. [1], by applying double immunofluorescence and immunohistochemistry on PCa and benign prostate hyperplasia biopsies, defined growth differentiation factor-15 (GDF-15) as an accurate biomarker to predict the aggressiveness and metastatic potential of PCa. They showed for the first time the increased frequencies of GDF-15+ cells alongside CD68+ M1- and CD163+ M2-macrophages, mostly in patients with high Gleason scores. They found such correlations not only intratumorally but also in extratumoral regions and in the luminal excrescences. Importantly, they found a preferential colocalization of GDF-15 and the presence of PD-L1 in luminal excrescences at all stages of PCa but with different patterns of expression, which were markedly increased in patients with Gleason score of nine. The authors conclude that the presence of GDF-15 in excrescences may also support its role as a non-invasive prognostic biomarker. One of the most common genetic alterations in PCa is the loss of phosphatase and the tensin homolog (PTEN) tumor suppressor gene, which is linked to advanced PCa progression and a poor clinical outcome. In their study, Cyll et al. [2] used convolution neural networks to develop a fully automated method for evaluating PTEN scores on immunohistochemistry-stained slides and to determine its prognostic value in PCa patients post-radical prostatectomy alone or combined with DNA ploidy status. The fully automated method correlated strongly with the established manual scoring. Using a discovery cohort and validating their results in an independent cohort, the authors demonstrated that PCa patients with PTEN-low had a statistically increased risk for biochemical recurrence post-radical prostatectomy versus those patients with PTEN-high. Importantly, this association remained significant in multivariate analyses, including routine clinicopathological parameters. Moreover, the combined analyses of fully automated PTEN and DNA ploidy status further improved risk stratification. Thus, this fully automated method, which was developed based on the use of convolution neural networks, could be applied for the accurate assessment of PTEN scores in immunohistochemistry slides, eventually replacing manual scoring, which is both time-consuming and, to some extent, subjective.

Recent advances in PCa translational research include the investigation of genomic aberrations that regulate disease progression and how these can be therapeutically targeted. As discussed above, the dysregulation of PTEN is often observed in primary prostate tumors which are characterized by few mutations as opposed to advanced prostate tumors which have more mutations. To this end, DNA damage repair (DDR) is often dysregulated in advanced PCa and associated with poor survival outcomes. In their work, Chang et al. [3] assessed the prognostic role of genetic variants in DDR pathways in patients with advanced PCa treated with androgen deprivation therapy (ADT). Of the various single nucleotide polymorphisms detected, one, *MSH2* rs1400633 C>G, was independently associated with both cancer-specific survival and overall survival (OS). Mechanistically, the MSH2 protein is involved in DNA repair pathways and, therefore, when mutated, *MSH2* may negatively affect this process and drive tumorigenesis. The authors showed that the presence of rs1400633 risk allele C increased MSH2 protein expression in the prostate and other tissues, and this was correlated with more aggressive disease. The findings of this study have important implications for the clinical treatment of PCa. Genotyping before ADT could help stratify PCa patients as high-risk for recurrence, thus being suggestive of more aggressive therapies, for instance, combining ADT with first-line chemotherapy or combining ADT with poly (ADP-ribose) polymerase (PARP) inhibitors given the sensitivity of DDR-deficient tumors to PARP inhibition. Radiotherapy (RT) has been shown to mediate immunomodulatory effects, leading to tumor regression not only locally, within the irradiation field, but also at distant metastatic sites, a phenomenon defined as the “abscopal effect”. The abscopal effect is at least partially mediated by T-cells within the tumor microenvironment which have been activated upon the recognition of tumor antigens released by the irradiated dying tumor cells and presented to them by professional antigen-presenting cells. Such tumor antigen-specific T-cells can reach metastatic sites via the bloodstream and lyse tumor cells. Thus, RT may act as an in vivo tumor vaccine. It becomes therefore conceivable that RT modulates patients’ T-cell repertoire. In the next review, my colleagues and I [4] discussed data from my laboratory which showed T-cell receptor (TCR) clonotype changes post-RT in patients with localized prostate cancer without any previous treatment. We detected the clonotype frequencies (CFs) of expanded and also contracted T-cell clonotypes as well as of new clonotypes. By performing a top 10 TCR Vβ CF ranking before and post-RT, we identified dynamic changes in the TCR repertoire which could serve as surrogate markers for response to treatment. More importantly, new clonotypes could be identified among patients with high Gleason scores, suggesting that the RT-induced remodeling of the TCR repertoire depends on tumor biology. These observations upgrade the role of RT in reinforcing antitumor immunity and propose its combined use with immunotherapies.

In the next study, Aubert et al. [5] proposed the CRISPR/Cas9-mediated deletion of prostate tumor-associated molecules considered to function as checkpoints, followed by the assessment of tumor growth in humanized mice, as a novel technological platform aiming at the discovery of novel immune checkpoints. To this end, they could demonstrate that the herpes virus entry mediator (HVEM), expressed by the prostate tumor, downregulates the functional program of CD8+ T lymphocytes via binding to its ligand, namely, the B- and T-lymphocyte attenuator (BTLA) molecule, thus facilitating prostate tumor growth in humanized animals. Blocking the interaction between HVEM and BTLA with an anti-HVEM monoclonal antibody resulted in (i) an increase in CD8+ T-lymphocyte proliferation; (ii) a reduction in the CD8+ T-exhausted phenotype; and (iii) a delayed prostate tumor cell-line growth in NOD.SCID.gc-null mice reconstituted with human T-cells. Moreover, by performing Cancer Genome Atlas (TCGA) analyses, the authors showed for the first time that HVEM and BTLA mRNA expression levels were strongly correlated with poor clinical outcomes in PCa patients, indicating an unfavorable prognostic role for the HVEM/BTLA immune checkpoint during PCa progression. In light of the disappointing clinical responses with anti-PD-1/anti-PD-L1 in PCa patients, the authors suggest that anti-HVEM therapy combined with immune checkpoint inhibition may enhance anti-tumor immunity in PCa.

Hasson et al. [6] highlighted the role of next-generation sequencing in the development of precision oncology approaches. They discussed that personalized targeted therapies can provide clinical benefits for cancer patients only when these are applied in the context of clinically relevant genomic alterations (CRGA). Comprehensive genomic profiling (CGP) may facilitate the detection of CRGA via the assessment of all classes of genomic alterations (GA) across a plethora of cancer-related genes. This was tested in a retrospective study which included a CGP group of ovarian cancer (OC) patients and a historical control group consisting of OC patients who were not referred to any genomic profiling. During a median follow-up time of almost 40 months, the clinical outcomes (OS and progression-free survival; PFS) were similar for the CGP and historical control groups. However, Cox regression analyses on the baseline parameters revealed a significantly longer median OS in the CGP group compared to the historical control group. By analyzing the CGP group, they found that patients who had received CPG-suggested therapies had a longer median OS as compared with the median OS of patients who did not have actionable mutations or were not provided with a CGP-suggested therapy. Among the molecular biomarkers analyzed, BRCA, CCNE1, and KRAS, as well as tumor mutational burden (TMB) and loss of heterozygosity (LOH), were predictive of OS. In particular, BRCA and high LOH were associated with a favorable prognosis to PARP inhibitors, whereas CCNE1 and KRAS were associated with a worse prognosis to platinum-based therapy. TMB (>4 mut/Mb) was indicative of a better prognosis in patients with OC. Therefore, CGP testing may provide more reliable prognostic and predictive insights for the treatment of OC and other types of cancer as well. Another type of gynecological cancer, namely, endometrial cancer (EC), is the most commonly diagnosed gynecologic neoplasm in Western countries. TCGA analyses introduced a molecular classification of EC in four groups, based on differences both in the genomic profile and in clinical outcomes (PFS), namely, the DNA polymerase epsilon (POLE) exonuclease domain-mutated, mismatch repair-deficient (MMR-D), p53-abnormal (p53abn) and no specific molecular profile (NSMP) EC groups, with the NSMP group being the most heterogeneous one, thus for which novel biomarkers of risk stratification are urgently needed. Ravaggi et al. [7] applied this TCGA molecular classification to a cohort of high-risk EC patients and investigated the expression of additional biomarkers useful for the further prognostic stratification of NSMP patients. They found that the expression of L1 cell adhesion molecule (L1CAM) was significantly associated with a poor outcome, regardless of the tumor grade and the FIGO stage. Thus, NSMP/L1CAM-positive EC patients exhibited an extremely poor prognosis and features of aggressive tumors, such as the absence of hormone receptors and the positivity of AT-rich interaction domain 1A (ARID1A). EC patients belonging to the NSMP group but being L1CAM-negative expressed estrogen and progesterone receptors and were ARID1A-negative; they showed better clinical outcomes. These data suggested that L1CAM expression may function as an unfavorable prognostic factor in high-risk NSMP EC. Given that L1CAM-positive platinum-treated patients relapse much faster than L1CAM-negative ones, it is possible that this biomarker is involved in the development of resistance to platinum-based chemotherapy.

The combined positive score (CPS) is the ratio of the combined expression of PD-L1 on tumor cells and immune cells to the number of all the tumor cells. The PD-L1 CPS identifies patients with gastric cancer (GC) who are most likely to respond to pembrolizumab therapy. In their retrospective study, Yu et al. [8] explored the relationship between different biomarkers, including an Epstein–Barr encoding region (EBER), microsatellite instability-high (MSI-H), and PD-L1, and clinical outcomes in patients with GC. Patients enrolled had received immune checkpoint inhibition (ICI) monotherapy, combined immunotherapy and chemotherapy, or combined immunotherapy. The analyses demonstrated that high PD-L1 CPS (≥5 or ≥10) and MSI-H functioned as independent favorable biomarkers predicting improved clinical responses to immunotherapies. Improved clinical benefits (overall response rate and PFS) were also observed in patients with EBER combined with CPS ≥ 1. In addition, the incidence of the combined expression of EBER or MSI-H and PD-L1 CPS ≥ 1 was high and associated with a better prognosis. Thus, the authors provided new results for the predictive role of MSI-H, EBER-positive, and PD-L1 for ICI in GC which, along with the proposed cut-off values, could provide the platform for the design of more efficient therapeutic treatments for GC patients. Genomic instability is linked to transcriptomic dysregulation in the tumor immune microenvironment with implications for prognosis, and the response to various treatments including chemotherapy, immunotherapy, and radiotherapy. Understanding the mechanisms underlying such alterations in the tumor microenvironment will lead to the discovery of novel therapeutic targets. Ye et al. [9] developed a multi-omics network to analyze integrated copy number variations (CNV) and gene expression in non-small cell lung cancer (NSCLC) tumors in order to unravel molecular networks that provide information on mechanistic pathways guiding the immunotherapeutic targeting of immune checkpoints, including CD27, PD-1, and PD-L1. The authors could identify a prognostic model consisting of five genes which was validated for patient stratification in TCGA databases. In the CD27, PD-1, and PD-L1 identified immune-omics network, a variety of genes were associated with sensitivity or resistance to chemotherapies, and also a response to radiotherapy in NSCLC cell lines and patient-derived tumors. The constructed immune-omics network revealed novel and promising therapeutic strategies providing the first evidence of the inhibitory effects of various cytotoxic drugs on the immune checkpoints CD27 and PD-1. The data from this study point to the complex elements comprising genes and proteins within the tumor immune microenvironment, which are critical for the immune status of the host–tumor interaction. Integrating these elements into an immune response score could help to select patients most likely to respond to immunotherapies.

The quality of resection in patients with sarcoma is a very important factor for disease-free survival (DFS), but there are no validated molecular biomarkers for prognosis or predicting responses to chemotherapy. Nassif et al. [10] explored the predictive role of molecular alterations identified in two large precision medicine trials, MOSCATO and ProfiLER, for responses to anthracycline-based chemotherapy. They reported that TP53 alterations were associated with shorter DFS and increased metastases, but also with better responses to anthracycline treatment. Thus, patients with localized sarcomas with TP53 mutations had mostly metastatic recurrences than loco-regional ones (93% vs. 7%, respectively), whereas there were 70% metastatic and 30% local relapses in the TP53 wild-type sarcomas. In addition, TP53 alterations were the only parameter that was significantly associated with DFS in a univariate analysis. In multivariate analyses, TP53 mutations, but not deletions (due to the low number of patients belonging to this group), were a significant prognostic factor in a model with various parameters, including histotype, grade, location, size, and type of therapy. Regarding the predictive value of TP53 mutations, the objective response rates to anthracycline-based chemotherapy were higher in patients with TP53-mutated sarcomas (*n* = 17) as compared to those observed in patients with wild-type TP53 (*n* = 125) (55% vs. 35%, respectively). This study had excessive inherent heterogeneity in terms of histotypes and, as the authors conclude, validation is needed in prospective studies with more homogenous histotypes for the study of the biomarker utility of various specific types of TP53 mutations.

There are doubts regarding the ideal cut-off for TMB as a predictive biomarker for selecting patients to respond to pembrolizumab-based therapies. Although the ≥10 mutations/Mb cut-off (TMB-high) has been used for the FDA approval of pembrolizumab for patients’ treatment, still a sizeable proportion of patients with TMB-high tumors do not respond to this type of ICI, making it necessary to identify additional molecular alterations that influence the TMB, which could assist in a better patient selection for immunotherapy. In this respect, the study by Xavier et al. [11] investigated the mutational profiles of patients from the study by Samstein et al. [12], which examined the association between TMB-high and survival outcomes in patients receiving ICI across a wide variety of cancer types. The authors could identify mutations in 27 genes which, in univariate analyses, correlated significantly with OS upon treatment with immune checkpoint inhibitors when tumors with TMB ≥ 10 mut/Mb were assessed. Among these, 5 mutated genes showed decreased OS (*STK11, KEAP1, CIC, E2F3*, and *TP53)*, whereas 22 mutated genes were associated with improved OS (*p* < 0.05 in both cases). Multivariate analyses confirmed the correlations in *STK11* and *E2F3* mutations with worse OS as well as those in *NTRK3, PTPRD, RNF43, TENT5C, TET1,* and *ZFHX3* with improved OS. This study highlights the role of specific somatic mutations in certain genes as predictive biomarkers, combined with TMB-high, for survival outcomes across several types of cancers.

Inflammasome activation via somatic mutations in hematopoietic stem and progenitor cells resulting in the activation of caspase-1 (Casp1) is key for abnormal hematopoiesis and inflammation in myelodysplastic syndrome (MDS). The activation of the inflammasome may also support tumor escape from immune surveillance via the increased expression of immune checkpoints (i.e., PD-1/PD-L1) [13,14], suggesting a promising role for immune-related biomarkers in the pathogenesis of MDS. However, the prognostic and diagnostic relevance of Casp1 and immune checkpoint molecules in MDS remains currently unclear. The article by Graf et al. [15] explored the use of the jointed analysis of Casp1 and PD-1/PD-L1 as potential immune-related diagnostic and prognostic biomarkers for MDS. They could demonstrate an inverse correlation with Casp1-high combined with PD-L1-low in lower-risk MDS patients compared with Casp1-low and PD-L1-high in higher-risk patients. Considering that Casp1-high/PD-L1-low is suggestive of immune activation, whereas Casp1-low/PD-L1-high associates with immune suppression, these findings may have a prognostic utility, proposing a dynamic progression of the disease from low-risk to high-risk based on the levels of an effective antitumor reactivity or of mechanisms promoting tumor immune escape. The utility of this discordant combined expression of Casp1/PD-L1 as a biomarker for MDS patients is strengthened by the finding that control groups with no inflammatory conditions, or with systemic inflammation, had concordant patterns of Casp1/PD-L1 expression (i.e., Casp1-low/PD-L1-low and Casp1-high/PD-L1-high, respectively).

Glioblastoma multiform (GBM) belongs to cancer types with extensive heterogeneity and a very poor prognosis. Apart from the anti-PD-1 treatment for patients with microsatellite instable (MSI) tumors, no immunotherapy is currently approved for GBM patients. Thus, the standard treatment for GMB consisting of concurrent RT and temozolomide-based chemotherapy remains the same for years now. In their review, Mensali and Inderberg [16] discusses various immunotherapeutic clinical studies, including ICI, therapeutic vaccinations, and administration of genetically engineered T-cells, which provided clinical benefits based on patients’ immune system activation. The lack of predictive biomarkers for this type of cancer provides a serious impetus for the selection of patients who could benefit from the appropriate therapy or from combined treatments. Immunosuppression within the tumor microenvironment (TME) due to increased frequencies of myeloid-derived suppressor cells, regulatory T-cells, and S100A4-positive suppressor macrophages are discussed as critical factors dampening tumor immune reactivity, thus promoting glioma growth. The authors also proposed the use of validated biomarkers, such as TMB, mismatch repair deficiency, and the PD-L1 expression level combined with novel emerging biomarkers linked to the immune TME to guide the selection of appropriate individualized therapeutic strategies and to improve the clinical benefits. In these lines, He et al. [17] proposed the therapeutic targeting of GBM stem-like cells (GSCs) via the discovery of GSCs specific markers. They have identified such markers using publicly available scRNA-seq data and by taking into account various reality-relevant parameters including the universality and significance of these markers, their unique expression in GSCs (and not normal brain cells), as well as the level of expression and their cellular location. They could identify one intracellular (TUBB3) and two cell-surface markers (PTPRS and GPR56) which fulfilled those criteria with the best scores and proposed them as potential markers for targeting GSCs in the context of therapeutic regimens for GBM.

Next, Karagiannakos et al. [18] discussed the importance of understanding the functional role of genomic alterations in cancer progression for the clinical application of efficient targeted therapies. To this end, they review signaling pathways that are deregulated in various types of malignancies and provide reports on corresponding therapeutic treatments. This review article provides important analyses of the mechanistic molecular events in cell signaling, which could have a significant impact on the identification of novel therapeutic targets, thus opening new avenues for the design of more efficacious therapeutic combinations. The next review by Fatima et al. [19] stresses the necessity of identifying predictive biomarkers in non-invasive liquid biopsies, which would be most useful for longitudinal studies for selecting patients with a high probability of responding to ICI. The authors mention that difficulties in frequently obtaining tumor biopsies along with tumor heterogeneity restrict the predictive reliability of traditional biomarkers. Their review has a focus on circulating tumor DNA, circulating tumor cells, immune cells and exosomes/extracellular vesicles, as well as soluble PD-1 and PD-L1, and provides representative current studies in which their predictive role was thoroughly explored. With the current predominance of ICI-based immunotherapeutic clinical trials, the authors expect a prominent increase in validation studies and peripheral blood-based assays as potential prognostic and predictive tools. The authors also comment that single biomarkers will not capture the entire picture of the molecular and cellular mechanisms underlying immune activation, clinical responses, or immune resistance during or post-immunotherapies and, therefore, multimodal approaches, including non-invasive biomarkers, will be required for an accurate prognosis and the monitoring of disease progression. Another non-invasive, soluble factor associated with cancer progression is Apelin, which is an endogenous ligand for the G protein-coupled apelin receptor and it is overexpressed in tumor tissues, particularly those metastasized. In their review, Grinstead and Yoon [20] supported an association of Apelin with clinical characteristics which ascribe a potential role for this molecule in predicting clinical treatment responses and prognosis. However, given the conflicting results relative to the quantification of Apelin serum levels in patients with different types of cancer and healthy donors, more studies will be required to provide solid conclusions for its prognostic utility. Regarding these conflicting results, the authors discuss that variations both in metabolic and physiologic processes among patients with various types of cancer may differently impact Apelin’s serum concentration. For more accurate measurements, they highlight the need to identify Apelin isoforms specific for certain patient populations, since the serum levels of the various isoforms of this molecule may vary among various cancer types. In this context, future studies will be more conclusive concerning the association between Apelin level changes in serum and disease progression for specific cancer types.

In their review, Ostrand-Rosenberg et al. [21] described the mechanisms underlying the accumulation and function of myeloid-derived suppressor cells (MDSCs) in cancer patients and being responsible for ineffective immunotherapies based mostly on ICI and genetically engineered T-cells. Such an increase in MDSCs activity can be considered to be an acquired immune resistance (AIR) mechanism developed by the tumors to decrease antitumor immunity. They discuss the essential role of the receptor for advanced glycation end-products (RAGE) and of its two ligands, S100A8/A9 and the high mobility group box protein 1 (HMGB1), in inducing accumulation and enhanced suppressor function of MDSCs. The authors note that the S100A8/A9 heterodimer in addition to RAGE also binds to TLR4 on the cell membrane to trigger a signal transduction pathway, resulting in the production of reactive oxygen species, nitric oxide, and several pro-inflammatory cytokines. So, presently, it is not known if S100A8/A9 and HMGB1 act solely via RAGE or whether the ligands alternatively bind to TLR4 to activate MDSCs. Moreover, the possibility exists that, besides RAGE, additional receptors within the TME may account for the activation of MDSCs. However, given the participation of RAGE in the early steps of the regulation of MDSCs’ development, its therapeutic targeting (also combined with HMGB1 and/or S100A8/A9 targeting) may significantly downregulate their suppressor function. Another AIR mechanism was described by my colleagues and myself [22]. Namely, we could show a direct correlation between the densities of cells with a cytotoxic effector phenotype (i.e., CD8+ cells) and densities of cells displaying suppressor phenotypes (i.e., CD163+ and FoxP3+ cells) in the primary tumors of patients with breast cancer. In the majority of the cases examined, we could demonstrate that cells with suppressor phenotypes are abundant in the tumor, only in the presence of high frequencies of cells with cytotoxic effector phenotypes. This is suggestive of an immune-intrinsic negative feedback mechanism of AIR, which is developed only during a robust endogenous antitumor immune response. Particularly, patients’ tumors with high CD8+ cell densities in both the tumor center (TC) and the invasive margin (IM) in their vast majority (almost 90%) also had high densities of either CD163+ cells or FoxP3+ cells in both regions. Importantly, tumors with low densities of CD8+ cells in both tumor regions were co-infiltrated by low numbers of CD163+ or FoxP3+ cells (in 85.4% of the cases examined). Interestingly, when analyzing the association between CD8+ cell frequencies with combined densities of CD163+ and FoxP3+ cells, a more profound expression of this infiltration profile was detected in that 100% of the tumors with CD8+ high densities in TC and IM also had high frequencies of CD163+ and FoxP3+ cells. On the other hand, none of the tumors with low CD8+ cell densities had high frequencies of CD163+ and FoxP3+ suppressor cells (all were infiltrated by low-density CD163+ and FoxP3+ cells). Similar results were obtained when the densities of CD8+ cells and of CD163+ or FoxP3+ cells were separately examined in TC or IM. Survival analyses showed a better OS for patients with low frequencies of CD163+ or FoxP3+ cells vs. patients with high frequencies of CD163+ or FoxP3+ cells, despite the fact that both groups had similar high frequencies of CD8+ cells. These results show that the increase in suppressor immune phenotypes intratumorally represents a negative feedback response to a robust endogenous antitumor immunity. They also propose that the quantification of CD8+ cells alone in the TME may be inaccurate for prognosis and should be analyzed along with cells displaying suppressor phenotypes.

This Special Issue included scientific studies (original research and review articles) which focused on the discovery of new highly sensitive and specific biomarkers for prognosis and prediction, risk stratification, and resistance mitigation, aiming at the development of personalized treatment schedules. The identification of such biomarkers in the tumor tissue and peripheral blood is mandatory for reliable diagnostics and the tailoring of therapeutic treatments which hold promise for further advancing the field of precision oncology. I sincerely hope that this comprehensive information on novel biomarkers for precision oncology will stimulate further translational and clinical research aiming at designing optimal therapeutic treatment protocols for specific cancer types, thus optimizing the efficacy of clinical trials.
